# Tropoelastin Promotes the Formation of Dense, Interconnected Endothelial Networks

**DOI:** 10.3390/biom11091318

**Published:** 2021-09-06

**Authors:** Aleen Al Halawani, Lea Abdulkhalek, Suzanne M. Mithieux, Anthony S. Weiss

**Affiliations:** 1 Charles Perkins Centre, The University of Sydney, Camperdown, NSW 2006, Australia; aleen.alhalawani@sydney.edu.au (A.A.H.); lea.abdul@gmail.com (L.A.); suzanne.mithieux@sydney.edu.au (S.M.M.); 2 School of Life and Environmental Sciences, The University of Sydney, Camperdown, NSW 2006, Australia; 3 Sydney Nano Institute, The University of Sydney, Camperdown, NSW 2006, Australia

**Keywords:** tropoelastin, angiogenesis, endothelial tube formation assay, mesenchymal stem cells, endothelial cells

## Abstract

Tropoelastin, the soluble precursor of elastin, has been used for regenerative and wound healing purposes and noted for its ability to accelerate wound repair by enhancing vascularization at the site of implantation. However, it is not clear whether these effects are directly due to the interaction of tropoelastin with endothelial cells or communicated to endothelial cells following interactions between tropoelastin and neighboring cells, such as mesenchymal stem cells (MSCs). We adapted an endothelial tube formation assay to model in vivo vascularization with the goal of exploring the stimulatory mechanism of tropoelastin. In the presence of tropoelastin, endothelial cells formed less tubes, with reduced spreading into capillary-like networks. In contrast, conditioned media from MSCs that had been cultured on tropoelastin enhanced the formation of more dense, complex, and interconnected endothelial tube networks. This pro-angiogenic effect of tropoelastin is mediated indirectly through the action of tropoelastin on co-cultured cells. We conclude that tropoelastin inhibits endothelial tube formation, and that this effect is reversed by pro-angiogenic crosstalk from tropoelastin-treated MSCs. Furthermore, we find that the known in vivo pro-angiogenic effects of tropoelastin can be modeled in vitro, highlighting the value of tropoelastin as an indirect mediator of angiogenesis.

## 1. Introduction

Angiogenesis involves branching of novel vasculature from existing vasculature at a wound site, subsequently giving rise to a capillary network that perfuses the affected region and restores circulation. The presence of blood vessels within the forming granulation tissue promotes cell viability and repair at the wound site, allowing diverse cells such as stem cells and fibroblasts to infiltrate and proliferate then deposit extracellular matrix. Viable blood flow is critical to biological repair for efficient tissue regeneration. This process is finely tuned, as demonstrated by an insufficiency of blood vessels during the latter stages of wound healing that can contribute to collagen accumulation and scar formation [1].

Capillary tube morphogenesis at the wound site is the process leading to spatial arrangement of functional capillary tubes [2]. This arrangement is in part governed by ECM components that could have either mitogenic or inhibitory effects, each working in concert to orchestrate capillary network formation. The main pro-morphogenic interstitial ECM components are fibrin, fibronectin, and collagen I [3]; however, tropoelastin—the precursor of elastin—has not been explored for its role in this process. Anti-morphogenic components are attributed to various ECM components such as basement membrane (BM) proteins deposited by the forming capillary networks as they achieve a mature and functional state, in order to shield endothelial cells and maintain network quiescence and integrity [4].

An endothelial tube formation assay (ETFA) is typically used to study angiogenesis in vitro [5,6,7]. ETFA relies on a basement membrane extract (BME) derived from Engelbreth-Holm-Swarm mouse sarcoma cells as a physical scaffold to support endothelial cell attachment and spreading into capillary-like networks. When endothelial cells are seeded in the presence of media containing pro-angiogenic factors they form traction centers by interacting with adhesion sites on the gel [8,9]. Subsequently, the endothelial cells physically interact with the underlying matrix to re-align the matrix fibers and drive endothelial cell tube formation.

The extent and complexity of the capillary-like networks are analyzed to assess the angiogenic potential of exogenous pharmacological or biological agents. Additionally, conditioned media from MSCs are enriched for pro-angiogenic factors [10]. There is interest in promoting these paracrine effects to enhance angiogenesis [11].

Beyond tropoelastin’s traditional role in elasticity as the monomer precursor of the ECM protein elastin, tropoelastin is now known to play a pivotal role in cell attachment, spreading, signaling, and tissue regeneration [12,13]. Cells in the ECM encounter tropoelastin during elastin synthesis. In contrast, in mature tissue there is limited elastogenesis so there is a corresponding drop in tropoelastin levels. However, injury of mature elastic tissue results in the proteolytic release of elastin fragments, whose cell-interactive sequences are functionally preserved in tropoelastin [13,14]. This accounts for tropoelastin’s signaling leading to the promotion of angiogenesis in vivo, but it is important to appreciate that it is unknown if endothelial cells respond directly to these elastic sequences or if the effect is mediated indirectly. The angiogenic potential of tropoelastin was first recognized in an in vivo study that utilized tropoelastin in a dermal regeneration template [15], and demonstrated an early angiogenic response to tropoelastin that contributed to wound healing in both mouse and porcine models. More recently, heat-stabilized pure tropoelastin implantable inserts were shown to enhance wound healing of full-thickness wounds in pigs, accompanied by evidence of perfusion in pink tissue that led to improved dermal growth and consequentially accelerated re-epithelialization [16]. These in vivo data from multiple animal models point to a consistent, yet poorly understood role for tropoelastin as a promoter of blood vessel formation.

An indication of tropoelastin’s ability to stimulate pro-angiogenic signaling emerged through the use of PLLA/PLGA scaffolds that were coated with tropoelastin. These scaffolds promoted mature vascular network formation in vitro with presumed cross-talk of co-cultured MSCs and endothelial cells [17]. Cytokine analysis performed on media collected from these co-cultured cells on tropoelastin-coated scaffolds revealed increased secretion of the angiogenesis-related cytokines HGF, VEGF, leptin, b-FGF, PIGF, ANGPT2, ANG, HB-EGF, PDGF-BB, and EGF. However, this co-culture system did not allow for dissection of the components and allocation to specific cells, nor could it exclude contributions due to complementary cell interactions that might promote and tailor vascularization.

In this study, we investigated interactions of tropoelastin with endothelial cells and MSCs, with the goal of distinguishing between direct and indirect aspects of tropoelastin-mediated angiogenesis. This approach allowed us to identify an inhibitory effect of tropoelastin on endothelial cells. In contrast, we found that the pro-angiogenic effects of tropoelastin are mediated through tropoelastin’s interaction with MSCs and over-ride the reluctance of endothelial cells to promote endothelial tube formation.

## 2. Materials and Methods

### 2.1. Cell Culture for HUVECs and MSCs

Human umbilical vein endothelial cells (HUVECs) (Lonza; C2517A, Basel, Switzerland) were seeded into cell culture flasks at a density of 2500 cells/cm^2^ and cultured at 37 °C, 5% CO_2_ in a humidified incubator in Endothelial Growth Medium-2 (EGM-2), which is endothelial cell basal medium (EBM-2) supplemented with 2% FBS, 0.4% hFGF-B, 0.1%VEGF, 0.1% R3-IGF-1, 0.04% hydrocortisone, 0.1% ascorbic acid, 0.1% heparin, 0.1% gentamycin and amphotericin B). On reaching 80–90% confluence at passages 6–8, cells were trypsinized and directly used for the tube formation assay. Human bone marrow-derived mesenchymal stem cells (MSCs) (ATCC; PCS-500-012, Manassas, VA, USA) were seeded into cell culture flasks at a density of 5000 cells/cm^2^ and cultured in Minimum Essential Media (α-MEM) supplemented with 10% (*v*/*v*) FBS (Life Technologies, Carlsbad, CA, USA), 2.4 mM l-glutamine (Lonza), and 1% (*v*/*v*) penicillin/streptomycin mixture at 37 °C, 5% CO_2_ in a humidified incubator. On reaching 80% confluence, cells were detached with a 0.05% (*v*/*v*) trypsin and 0.02% (*v*/*v*) EDTA which was neutralized with three volumes of α-MEM and centrifuged at 900× rpm for 5 min. Cells were resuspended in α-MEM and cultured in uncoated and tropoelastin-coated wells to produce conditioned media.

### 2.2. Cell Proliferation Assays

Four wells per 96-well plate were coated with 200 μL 20 μg/mL tropoelastin for 16 h at 4 °C then washed with PBS. Cells (HUVECs or MSCs) were seeded in 96-well plates at 1600 cells/well (5000 cells/cm^2^) on uncoated or tropoelastin coated (substrate-bound) wells in quadruplicate. At 1, 3, 5, and 7 days, media were removed and plates were washed with PBS for CyQUANT (ThermoFisher Scientific, Waltham, MA, USA) cell proliferation analysis.

### 2.3. Endothelial Tube Formation Assay with Tropoelastin and Conditioned Media

Where indicated, the tube formation assay was performed on 96-well plates coated with 50 µL basement membrane extract (Cultrex BME RGF; R&D systems, Minneapolis, MN, USA) after which HUVECs were seeded at 15,000 cells/100 μL. All other tube formation assays were performed on angiogenesis microslides (Ibidi, Gräfelfing, Germany) where 10 µL BME was coated on the inner well, and 11,000 cells/50 µL media were added to the upper chamber. ETFA was performed either in the presence of soluble tropoelastin (0.2 µg/mL; 96-well plates) or conditioned media from MSCs cultured on plates ± tropoelastin (TE) coating (20 µg/mL for 16 h at 4 °C; Ibidi microslides). Conditioned media (CM) was prepared by culturing MSCs on uncoated and tropoelastin-coated 6-well tissue culture plates. Media were conditioned over 48 h then collected from the tropoelastin-coated (CM+TE) and uncoated wells (CM-TE) and centrifuged at 3000× *g* for 10 min. The supernatant was transferred to 3 kDa cut-off centrifugal filters (Amicon Ultra; Merck, Burlington, MA, USA) for concentration for 60 min at 5000× *g*. For ETFA, pre-confluent HUVECs were detached, and trypsin was neutralized with three volumes of EGM-2. Cells were centrifuged at 200× *g* for 3 min and the supernatant was discarded. The remaining pellet was resuspended in an appropriate volume of EBM-2 for counting. For experiments testing the direct effect of tropoelastin on endothelial tube formation, the final cell seeding concentrations (15,000 cells/50 µL) were adjusted using EBM-2 only or EBM-2 supplemented with 0.2 µg/mL tropoelastin. For conditioned media experiments using Ibidi microslides, cells were counted and equally distributed among four empty tubes corresponding to different experimental conditions and 1 mL respective media (EGM-2, EBM-2, CM-TE, CM+TE) was added to further dilute out possible residual media. After centrifugation, supernatant was discarded and an appropriate volume of the desired media was added to each tube such that the final concentration was 11,000 cells/50 μL media, corresponding to the maximum volume of Ibidi microslide upper chambers (50 μL). 1% CM was used (100× dilution in EBM-2) in all tube formation experiments using conditioned media. Live cell imaging used a Nikon spinning-disk confocal microscope, with images taken every 1 h over 15 h using a 4× objective and brightfield (BF) microscopy.

**Figure A1:**
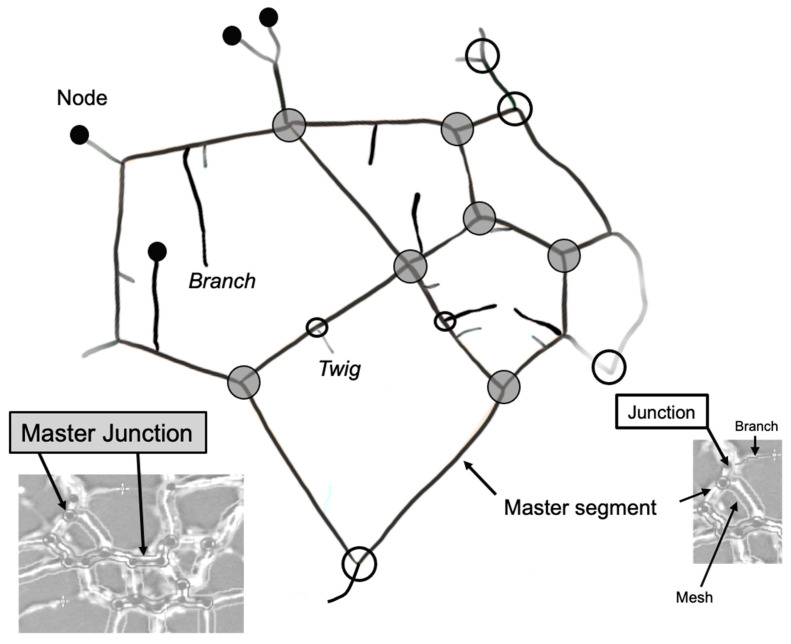
Angiogenic features of interest quantified by the plugin and the degree of their contribution to network complexity.

During nascent tube formation, HUVECs protrude from clustered cells and tubes and initially form branches. Subsequently, branching cells assemble to form an enclosed network of tubes. To assay for a pro-angiogenic response, network formation was assessed by tube and mesh formation. Further analysis measured the number of segments, master segments, enclosed meshes, and total tube length, which is the total length of segments, master segments, and branches within a well (Appendix A Figure A1 and Table A1). Analysis of the tube network complexity was performed by quantifying master junctions and master segments, which provided insight into the spatial arrangement of meshes.

### 2.4. Analysis and Statistics

Brightfield images were inverted on Image J and analyzed using the ImageJ Angiogenesis Analyzer Plugin (http://image.bio.methods.free.fr/ImageJ/?Angiogenesis-Analyzer-for-ImageJ accessed on 5 September 2021) to quantify branches, segments, master segments, meshes, master junctions, and mesh indices. Total tube length in pixels was calculated by adding branch, segment, and master segment lengths. Two-way repeated measures ANOVA was performed for all proliferation and tube formation assays. Following two-way ANOVA, Fisher least significant difference test was performed on tube formation data comparing CM-TE and CM+TE [7].

## 3. Results

### 3.1. Endothelial Cell Proliferation Is Unaffected but Mesenchymal Stem Cell Proliferation Increases in the Presence of Tropoelastin

**Figure 1:**
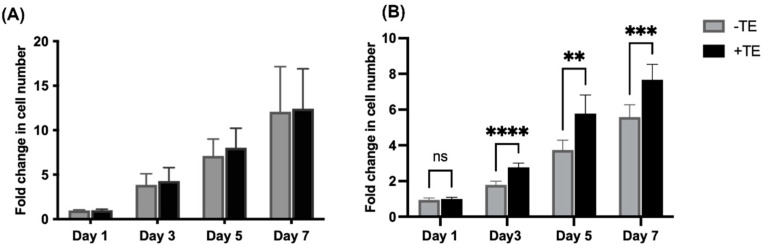
Effect of tropoelastin (TE) on cell proliferation. (**A**) HUVECs and (**B**) MSCs were cultured in the absence (gray) or presence (black) of adsorbed tropoelastin over a seven-day period. Fold changes in cell number were normalized to day 1 and at each time point fold change was compared between -TE and +TE. Asterisks show ** *p* < 0.01, *** *p* < 0.001, **** *p* < 0.0001. ns = not significant. Bars represent SD.

Cell proliferation assays were performed on HUVECs and MSCs to explore the effects of tropoelastin on proliferation. The cells were cultured on uncoated, or tropoelastin-coated TCP and fold changes were calculated by normalizing cell numbers at days 3, 5, and 7 post-seeding to cell numbers at day 1. No significant difference in proliferation rate was found between HUVECs grown in the presence or absence of tropoelastin (Figure 1A). In contrast, increased MSC numbers were observed on tropoelastin as early as day 3. These differences persisted over days 5 and 7 with a greater degree of MSC expansion occurring in tropoelastin-coated wells (Figure 1B).

### 3.2. Tropoelastin Impedes Endothelial Network Formation

**Figure 2:**
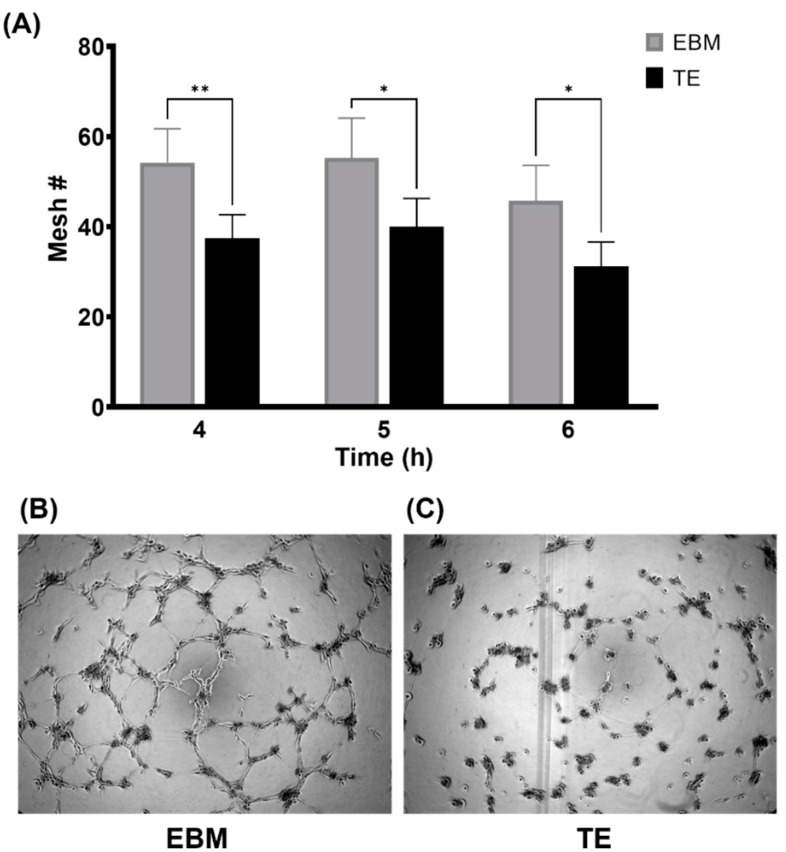
Direct use of tropoelastin (TE) impeded tube formation. (**A**) Quantitative summary of mesh numbers (Mesh #) in the absence (gray) or presence (black) of 0.2 µg/mL tropoelastin at 4, 5, and 6 h post-seeding. Tube formation assay was performed in a 96-well plate and images were taken at 6 h post-seeding. Representative tube formation in (**B**) EBM (referring to EBM-2) and (**C**) EBM + tropoelastin. Asterisks show * *p* < 0.05, ** *p* < 0.01. Bars represent SD.

As meshes represent enclosed networks of endothelial tubes, manual mesh counts were assessed and found to be significantly lower when tropoelastin was added to EBM-2 (Figure 2A). Phase contrast images showed a lack of tube formation in the presence of 0.2 µg/mL tropoelastin (Figure 2C) at the 6 h time point compared to the negative control (Figure 2B).

### 3.3. Conditioned Media from MSCs Cultured on Tropoelastin Results in Dense Interconnected Networks

**Figure 3:**
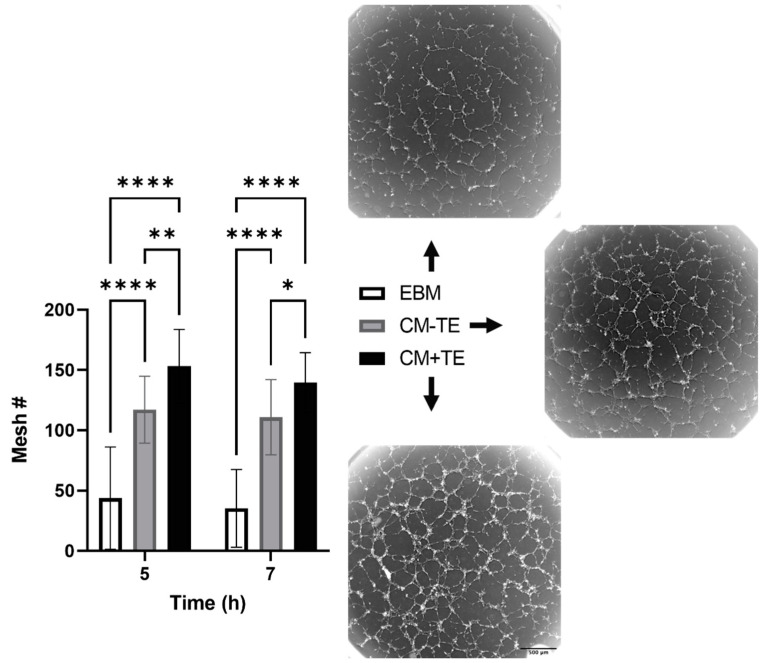
Mesh formation using conditioned media. Mesh numbers (Mesh#) were quantified at 5 and 7 h post seeding. Data were analyzed from six independent experiments with at least triplicate measurements per condition per experiment. Images show 5 h post-seeding, corresponding to peak tube formation. EBM; endothelial basal medium, CM-TE; conditioned media from tropoelastin-free MSC cultures, CM+TE; conditioned media from MSCs cultured on surface-bound tropoelastin. Asterisks represent statistically significant differences between conditions * *p* < 0.05, ** *p* < 0.01, **** *p* < 0.0001. Bars represent SD.

Conditioned media (CM) were collected from MSCs cultured on either uncoated (CM-TE) or tropoelastin-coated (CM+TE) 6-well plates, then used in ETFA. CM from both conditions elicited a pro-angiogenic effect, with significant increases in mesh numbers compared to EBM. The number of meshes formed in CM+TE at 5 and 7 h were higher than those formed in CM-TE (Figure 3). Mesh numbers in CM+TE were comparable to those observed in EGM-2 (not shown), a medium that contains pro-angiogenic factors.

### 3.4. Endothelial Tube Branching and Anastomosis

**Figure 4:**
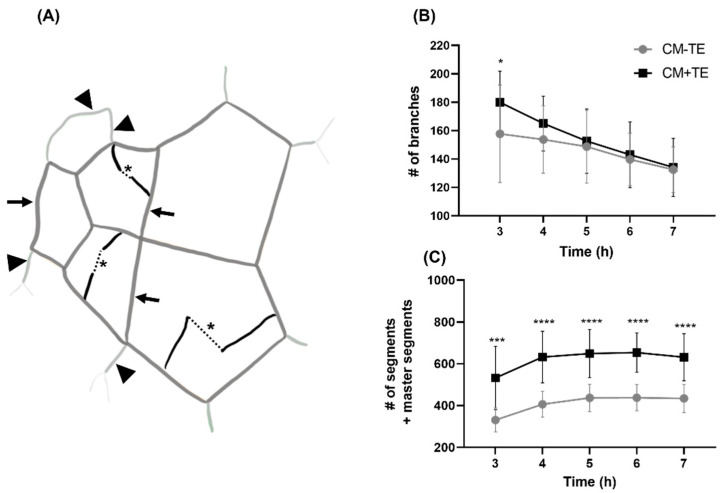
Effect of conditioned media from mesenchymal stem cells cultured in the presence (black) or absence (gray) of tropoelastin (TE) on endothelial tube branching and anastomosis. (**A**) Branches are depicted in bold black lines in schematic and dotted lines demonstrate anastomosis (*) of these branches to eventually become segments (large arrow heads) or master segments (arrows). (**B**) The number of branches (# of branches) and (**C**) segments and master segments (# of segments and master segments) over a 7 h period from five independent experiments with at least triplicate measurements per condition per experiment. All results shown are from conditioned media concentrated using 3 kDa cut-off centrifugal filters. * *p* < 0.05, *** *p* < 0.001, **** *p* < 0.0001. Bars represent SD.

EFTA features such as segments and master segments mostly form mesh boundaries. Master segments primarily form meshes within the center of the networks whereas segments, in conjunction with master segments, are implicated in mesh formation towards the periphery. Although segments and master segments form enclosed meshes, these two features are made possible by branch anastomosis: cells that branch off tubes eventually anastomose with neighboring branches to become segments or master segments. Dotted lines in Figure 4A represent the anastomosis of these branches. The complexity of the tubes formed, such as segments or master segments, and eventually the complexity of the network is determined by where anastomosis occurs. For example, if anastomosis occurs within an existing mesh, this divides one mesh into two or more, giving rise to a more complex mesh spatial arrangement. If anastomosis occurs towards the periphery of the network, it can result in either a new mesh or a longer tube being formed. If branches fail to anastomose, this can result in a less dense network with less meshes due to the lower number of segments and master segments. Branching at 3 h post-seeding was significantly higher in HUVECs treated with CM+TE (Figure 4B). Over time, there was a decrease for CM+TE showing a marked decrease in the number of branches. This rapid decline in the number of branches, in conjunction with Figure 4C showing a greater increase in the number of segments and master segments, reveals that conditioned media obtained from MSCs cultured in the presence of tropoelastin encouraged rapid branch anastomosis. This anastomosis comprised segments and/or master segments.

**Figure 5:**
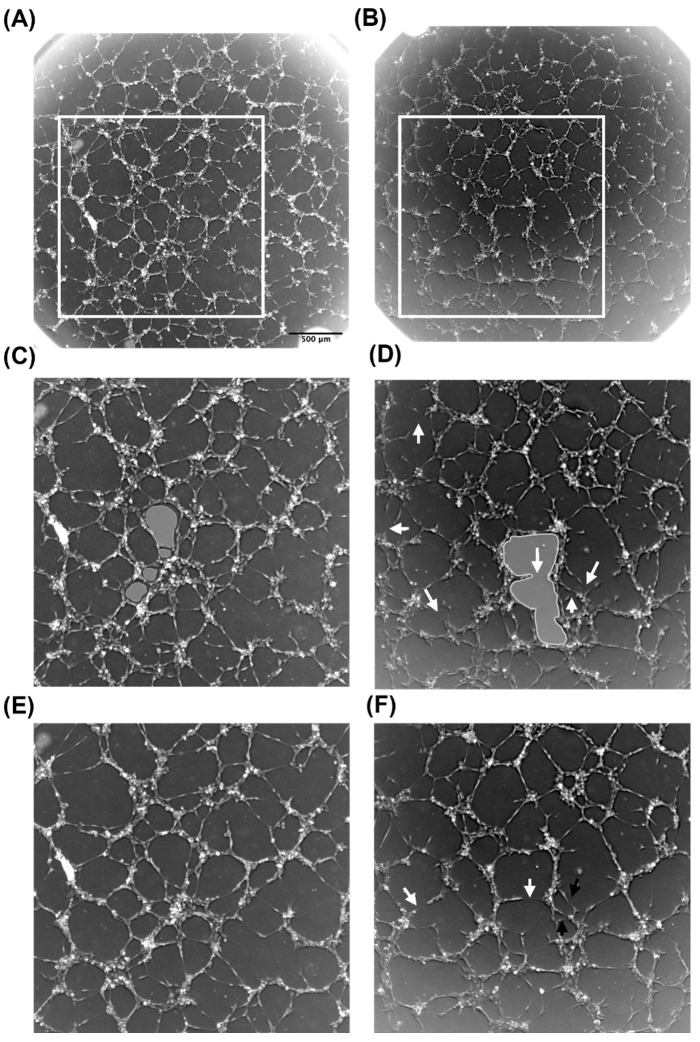
Effect of tropoelastin-based conditioned media from MSC cultures on endothelial network branching and mesh formation. Networks forming at 5 h in the presence of conditioned media from MSCs cultured with (**A**) or without (**B**) tropoelastin. Panels (**C**,**D**) are magnified sections from (**A**,**B**). Panels (**E**,**F**) represent these networks at 7 h. White arrows point to spaces formed by branches that had not anastomosed to form continuous segments. Black arrows point to an example of open areas that were enclosed by 7 h. Gray-shaded areas highlight differences in mesh characteristics.

Branches that formed in the presence of CM+TE rapidly anastomose and contribute to mesh formation. Multiple meshes formed at 5 h post-seeding in the presence of CM+TE within an outlined area of the network (Figure 5A) compared to a more open and spacious mesh formed in CM-TE (Figure 5B). Some branches had not anastomosed at 5 h but continued to anastomose by 7 h (black arrows Figure 5F), whereas networks in CM+TE were mostly enclosed by 5 h. Anastomosis of some branches was countered by some master segment and segment breakage, consistent with the nature of the slow decline in branches (Figure 4B) in CM-TE. These results reveal that networks formed in the presence of conditioned media from tropoelastin treated MSCs are denser and more complex. To further explore this finding, mesh index, master junction density, and total tube length were quantified.

### 3.5. Network Complexity and Interconnectivity

**Figure 6:**
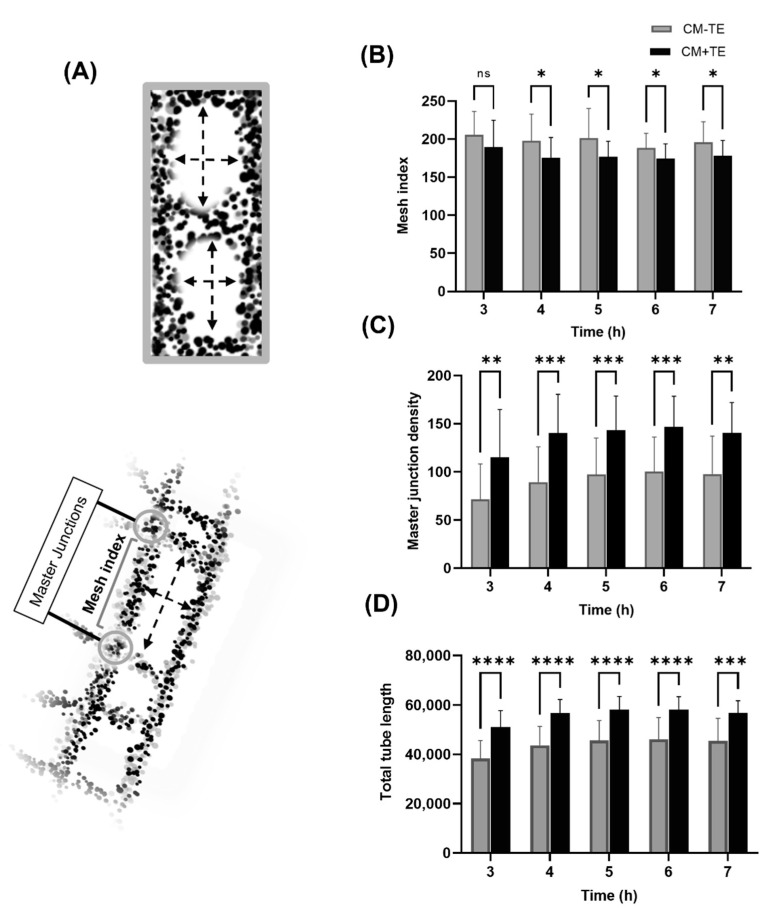
Effect of conditioned media on network complexity and interconnectivity. (**A**) Schematic showing mesh index and master junctions. The top diagram represents a small mesh index and shows close master junctions, while the lower diagram displays master junctions that are further apart. (**B**) Mesh index over time in conditioned media from MSCs cultured without (gray) or with (black) tropoelastin. (**C**) Master junction density over time in conditioned media from MSCs cultured without (gray) or with (black) tropoelastin. (**D**) Tube length refers to the sum of branch, segment, and master segment lengths from 3 to 7 h post-seeding in conditioned media from MSCs cultured without (gray) or with (black) tropoelastin. * *p* < 0.05, ** *p* < 0.01, *** *p* < 0.001, **** *p* < 0.0001. ns = not significant. Bars represent SD.

The mesh index measures the distance (in pixels) separating two master junctions (Figure 6A). Master junctions join three master segments (Appendix A Figure A1 and Table A1) providing a higher degree of complexity and interconnectivity to the network due to adjacent formed meshes. High mesh numbers can arise if the master junction density is high and mesh index is low within a network. The mesh index was reduced in CM+TE, meaning that the distance separating master junctions, hence the length of a master segment per formed mesh, was less (Figure 6B) implying smaller mesh boundaries and meshes. Also, the master junction density from 4 to 7 h was higher in CM+TE (Figure 6C).

Total tube length represents the total length of segments, master segments and branches combined, excluding elements that are not directly implicated with the formed network. The total tube length was significantly higher in CM+TE, due to an increase in the extent of tube formation and greater total path length of the formed tubes (Figure 6D). These data establish that networks that form in the presence of CM+TE are denser and exhibit a higher degree of complexity.

## 4. Discussion

We found that tropoelastin’s promotion of endothelial tube formation in vitro mirrors the in vivo pro-angiogenic effects of tropoelastin. There are two aspects of this process: (1) tropoelastin inhibits the action of endothelial cells directly, and (2) tropoelastin promotes angiogenesis through MSCs that in turn communicate the resulting pro-angiogenenic signal to endothelial cells. The angiogenic effect was magnified when HUVECs were treated with CM+TE where HUVECs exhibited rapid and abundant branching from nascent tubes. As tropoelastin indirectly enhances angiogenesis through support cells such as MSCs, it is worth noting that the number of branches rapidly declined in the presence of CM+TE compared to CM-TE. This decline correlated with an increased number of segments and master segments that persisted over 7 h. In both conditions, the decline in the number of branches coincided with accumulating segments and master segments, while significantly higher numbers were noted in the presence of CM+TE from 3 to 7 h, as expected for branches that rapidly anastomose and participate in mesh formation as segments or master segments.

In vivo, capillary networks that originate from arterioles undergo gradual bifurcations to terminate at multiple venule points, forming an enclosed and continuous network that ensures continuous blood flow. This is modeled in the in vitro study here, where the enclosed meshes formed by HUVECs resemble capillary beds with varying degrees of complexity, referring to complex network geometries portrayed in the proximity of meshes. More meshes forming back-to-back represent a more interconnected and complex network. This was shown by the master junction density and mesh index where the mesh index was significantly lower in the presence of CM+TE arising from shorter master segments. While master segments and segments are implicated in complex network formation, it is the number of master junctions that is indicative of network complexity [18]. As master junctions only join master segments, the number of master junctions correlated with the number of master segments, both of which were higher in the presence of CM+TE.

The lengths of master segments were shorter in the presence of CM+TE, as indicated by the mesh index, while both the number of master segments and segments were higher and the total tube length was greater, resulting in a greater surface area occupied by the network. This mimics what is seen for capillary networks in vivo where a dense capillary network in vivo allows for rapid gas and nutrient exchange between cells and capillaries, as the surface area available for diffusion in vivo is proportional to the number of perfused capillaries [19]. If meshes are dispersed such as that seen with greater distance between master junctions, nutrient and gas exchange are impeded by their diffusion limit so only cells in close proximity to the vessel boundaries survive, leaving the center susceptible to necrosis. Resulting from tropoelastin’s beneficial effects, these dense networks help to rapidly perfuse tissue in order to promote wound healing and repair.

## 5. Conclusions

The pro-angiogenic activity of tropoelastin is not mediated through direct effects on endothelial cells. Tropoelastin acts through participating MSCs, which in turn transmit pro-angiogenic signals to endothelial cells. Tropoelastin alters the secretory profile of MSCs to establish a pro-angiogenic environment in vitro. This effect can be separated mechanistically with conditioned media from MSCs that had been cultured on tropoelastin. These findings highlight the indirect role of tropoelastin as a signaling molecule in promoting angiogenic responses and demonstrate the benefit of incorporating tropoelastin into scaffolds to promote vascularization.

## Data Availability

The data presented in this study are available on request from the corresponding author. The data are not publicly available due to postgraduate thesis considerations.

## Appendix A

**Table A1:** Definitions of quantified Angiogenic Features.

**Master Segment ***	Tubes delimited by two junctions or master junctions that are further implicated with other segments and/or branches.
**Master Junction ***	Junction linking at least three master segments.
**Segment ***	Delimited by two junctions.
**Junction ***	Links two master segments as well as segments. Contribute to the non-free end of a branch or a twig.
**Branch ***	Tube delimited by a junction and a node. A branch is identified by the plugin as so if its length meets a set threshold value.
**Twig**	A short branch that is below the threshold cut-off for what the plugin quantifies as a branch.
**Node**	Free end of a branch or a twig. Also present where master junctions and junctions are detected.

* Refers to features analyzed.
